# Supplementary material for: Chromosomal instability by mutations in the novel minor spliceosome component CENATAC
Source: EMBO J. 2021 May 19;40(14):e106536. doi: 10.15252/embj.2020106536 (PMC8280824; doi:10.15252/embj.2020106536)
Supplement: Supplementary file 1 — Appendix [file EMBJ-40-e106536-s009.pdf]

Appendix Figures S1-S13

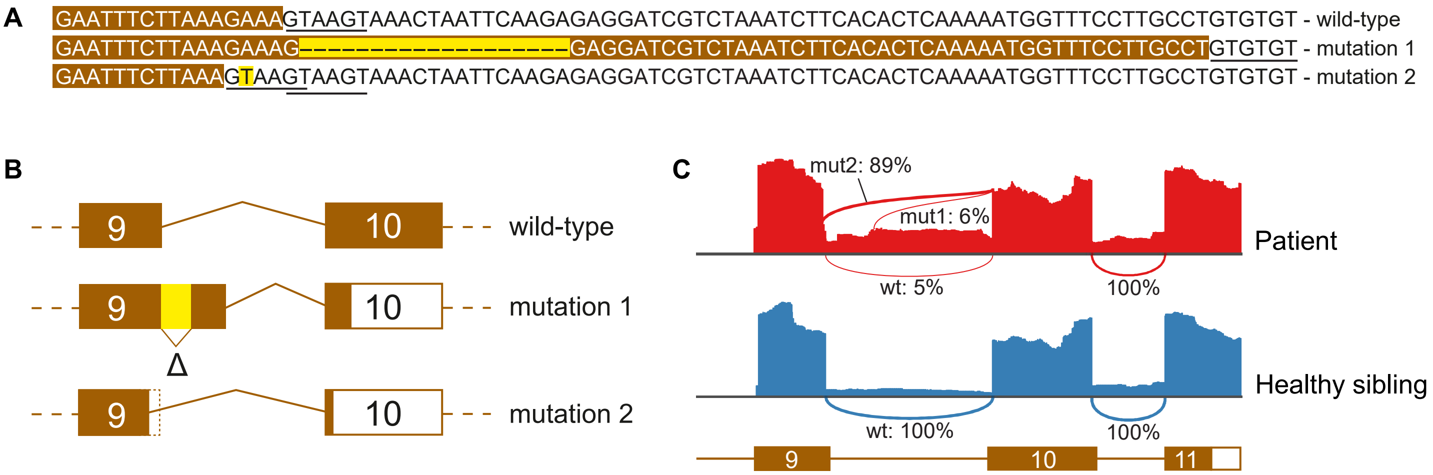

Appendix Figure S1. The effect of the MVA patient mutations on splicing of *CENATAC* intron 9. **A)** DNA sequences of exon 9 (white in brown boxes) and intron 9 (black) of wild-type and MVA mutated *CENATAC* alleles. The mutations (c.805+2_805+19del18, mut1; and c.803A>T, mut2) are indicated with yellow. Both the new and original 5' splice sites are underlined. Note that the original 5' splice site is lost in MVA mutant 1 but it is retained in MVA mutant 2. **B)** Schematic showing the effect of the MVA patient mutations on splicing of CENATAC intron 9. Exons 9 and 10 are represented with rectangles; protein-coding regions within exons are indicated with fill, untranslated regions without fill. **C)** Sashimi plots derived from patient and healthy sibling RNAseq data showing showing the effect of the MVA patient mutations on 5' splice site useage (percentages) of intron 9 in patient cells. The different splice sites are shown in A).

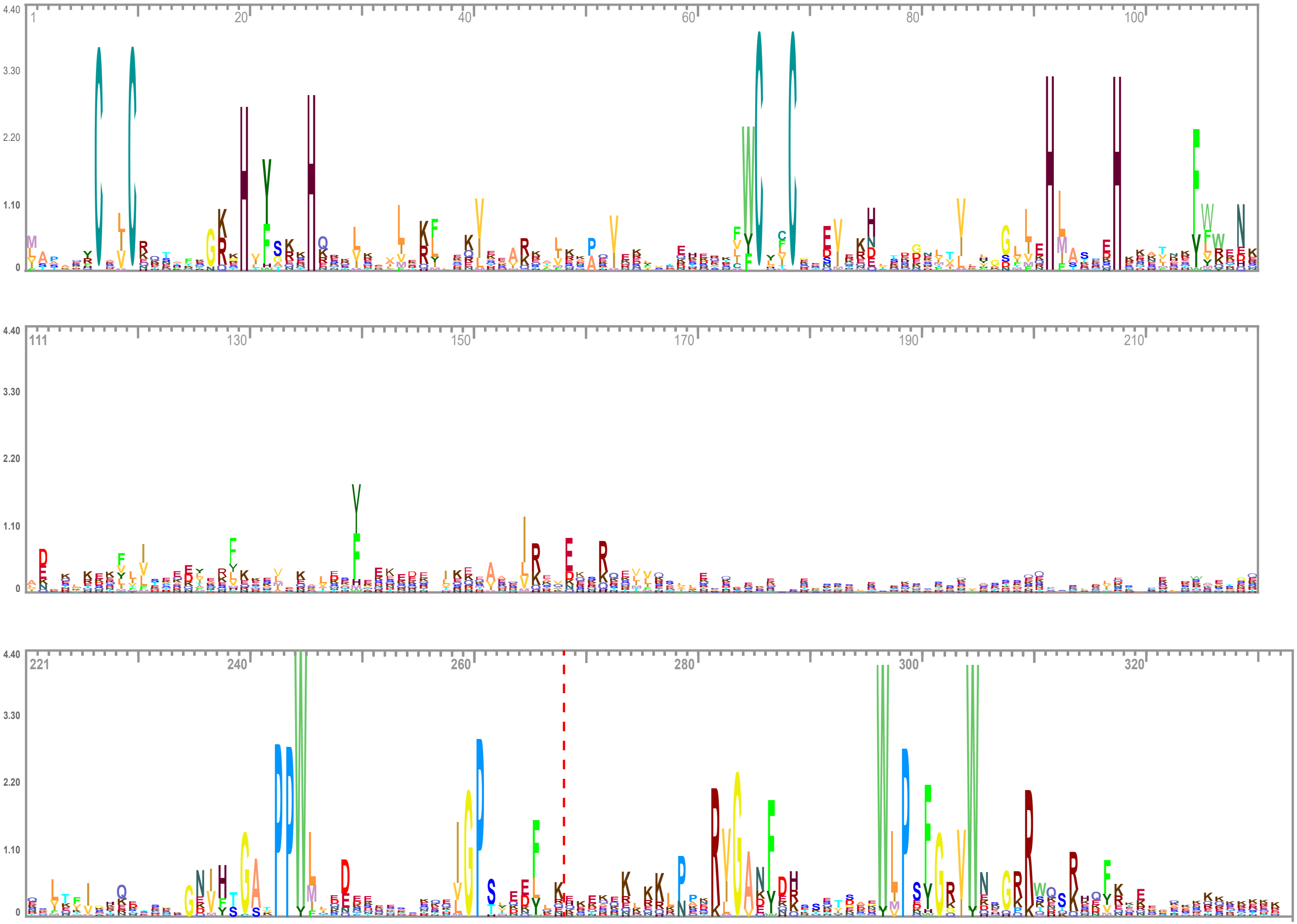

Appendix Figure S2. Full-length sequence logo of CENATAC's conserved residues (in metazoan species) split in three. Amino acids are numbered. The location of the truncating MVA mutations is indicated with the red dotted line.

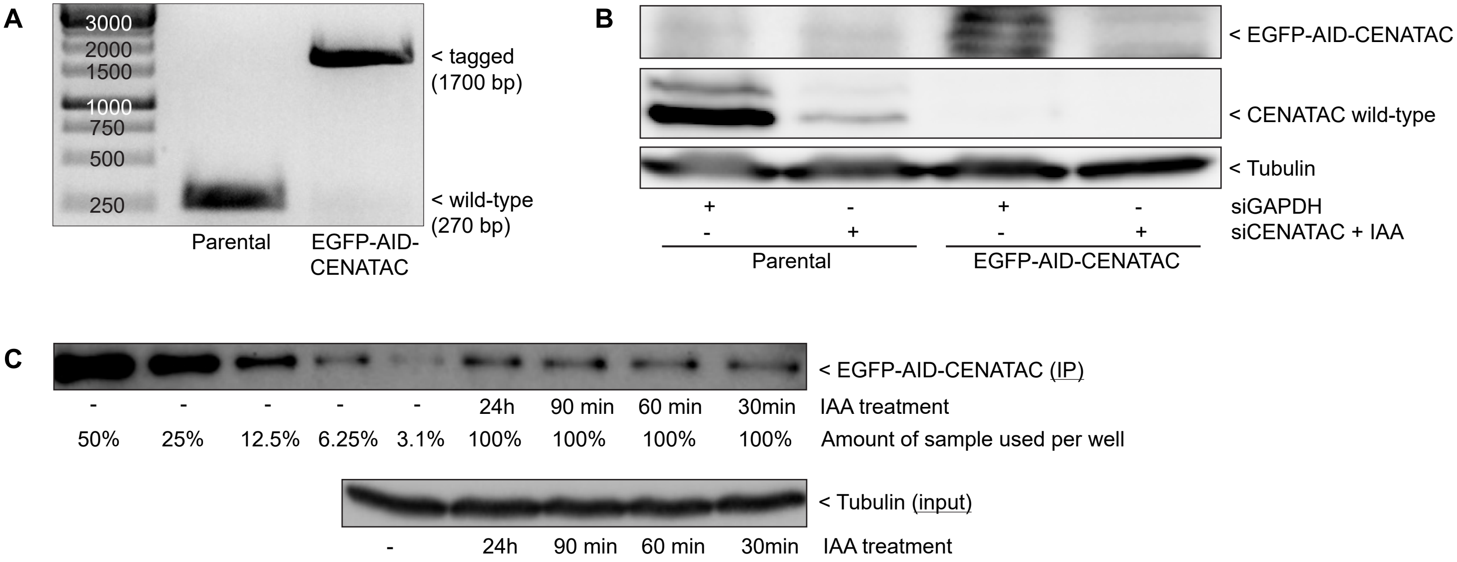

Appendix Figure S3. Endogenous EGFP-AID-CENATAC tag verification and CENATAC depletion. **A)** Genomic PCR of the CENATAC locus around the start codon. Left: parental cells; right: EGFP-AID-CENATAC cells. **B)** CENATAC (αCENATAC) and tubulin (αTubulin) immunoblots of EGFP-AID-CENATAC and parental cells treated as indicated for 48 hours. **C)** CENATAC (αCENATAC) immunoblot of EGFP-AID-CENATAC immunoprecipitated from cells treated with IAA for the indicated amounts of time (upper) and Tubulin (αTubulin) immunoblot of input samples (lower). The untreated IP sample (upper gel, 5 left-most wells) was divided into volumes of 50, 25, 12.5, 6.25 and 3.1% of the totoal volume, as indicated, for comparison with the IAA treated samples (4 right-most wells) of which 100% of the total volume was loaded. IAA, 3-indoleacetic acid.

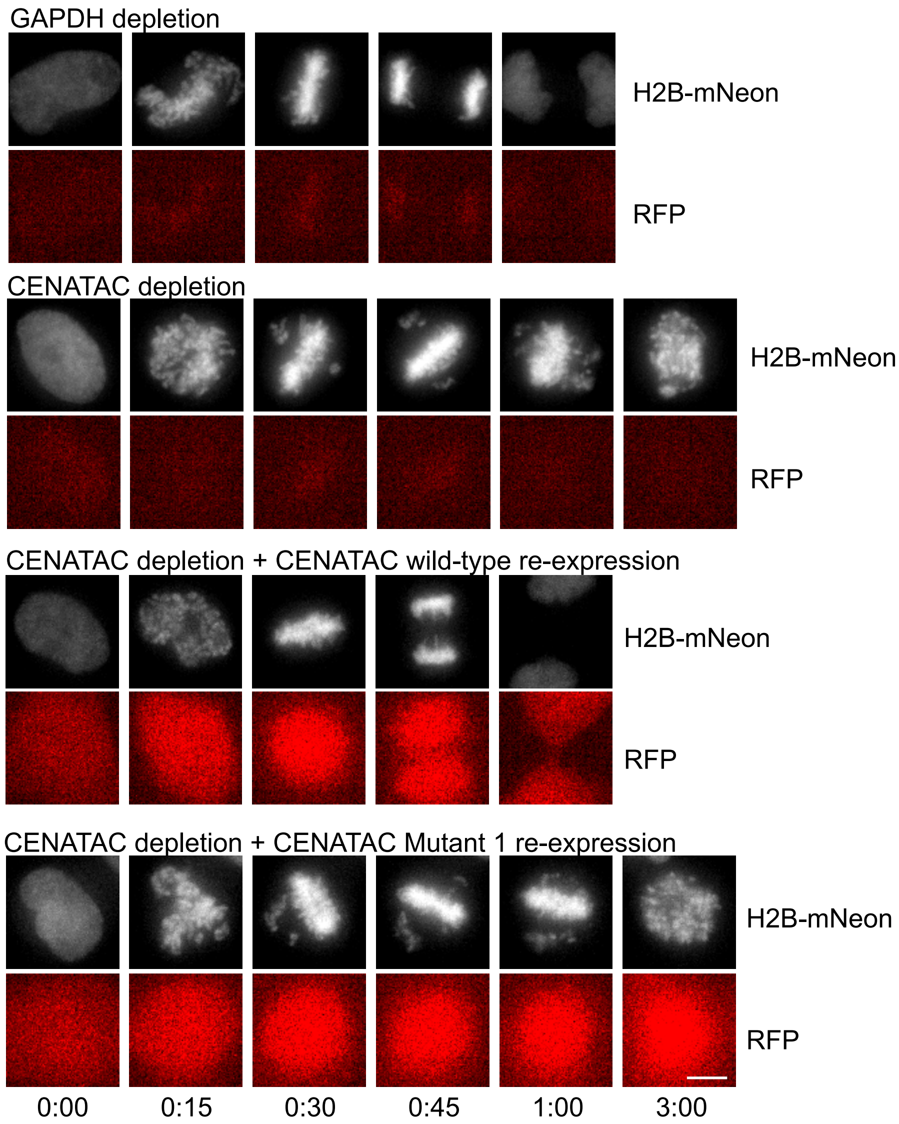

Appendix Figure S4. EGFP-AID-CENATAC cells expressing H2B-mNeon depleted of GAPDH or CENATAC, with or without re-expression of CENATAC variants as indicated (as in Fig. 2B-C). Expression of cytosolic RFP was used as a marker to identify cells expressing CENATAC variants. Upper panels: H2B-mNeon; lower panels: RFP. Time in hours. Scale bar, 10 µm.

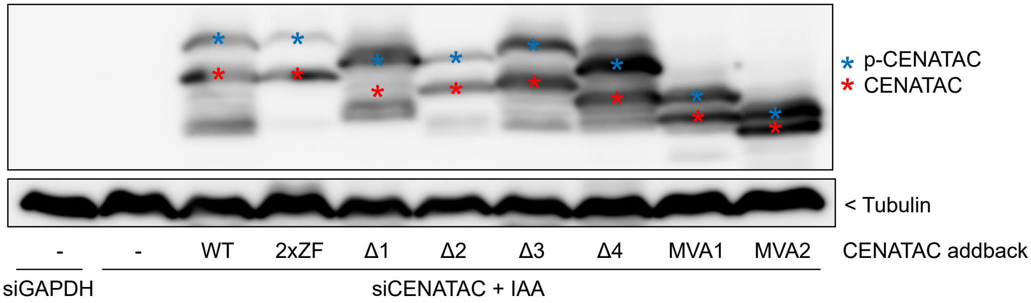

Appendix Figure S5. CENATAC (αCENATAC) and tubulin (αTubulin) immunoblots of cells treated as in Fig. 2C. p-CENATAC indicates phosphorylated CENATAC (blue asterisks). IAA, 3-indoleacetic acid.

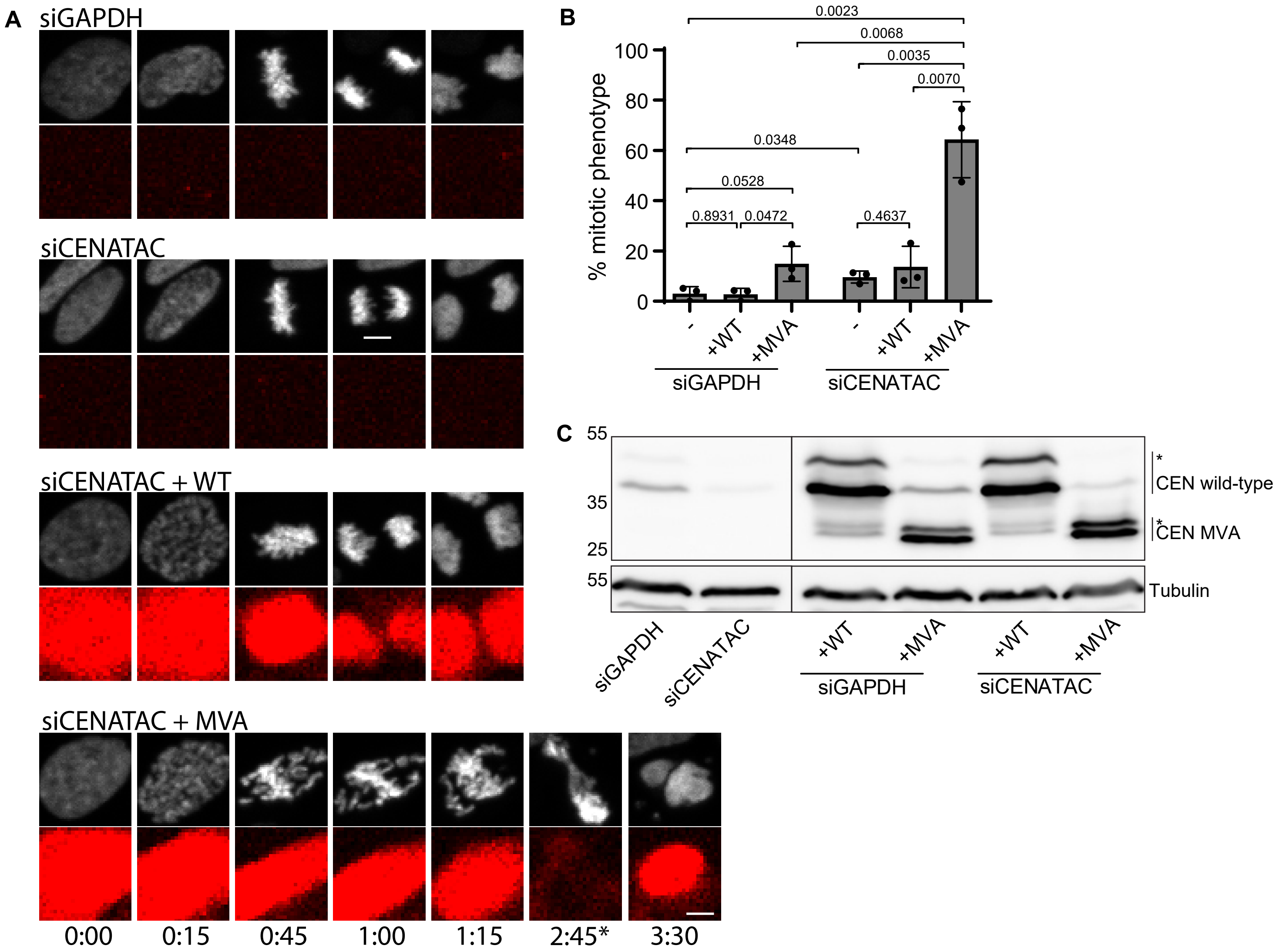

Appendix Figure S6. CENATAC depletion and re-expression in DLD-1 cells. **A)** DLD-1 cells expressing H2B-mNeon, depleted of GAPDH or CENATAC, with or without re-expression of CENATAC wild-type (WT) or MVA mutant 2 (MVA) as indicated. Expression of cytosolic RFP was used as a marker to identify cells expressing CENATAC variants. Upper panels: H2B-mNeon; lower panels: RFP. Time in hours. Scale bar, 10 µm. *The RFP signal is out of focus here. **B)** Quantification of mitotic defects as in A) of H2B-mNeon-expressing DLD-1 cells treated as indicated. Each bar depicts the mean of three independent experiments ± s.e.m., with >54 cells in total per condition. P values were calculated with two-sided unpaired Student's t tests. **C)** CENATAC (αCENATAC) and tubulin (αTubulin) immunoblots of DLD-1 cells treated as in B). CEN, CENATAC; MVA, MVA mutant 2 (Fig 1). Phosphorylated CENATAC is indicated with asterisks.

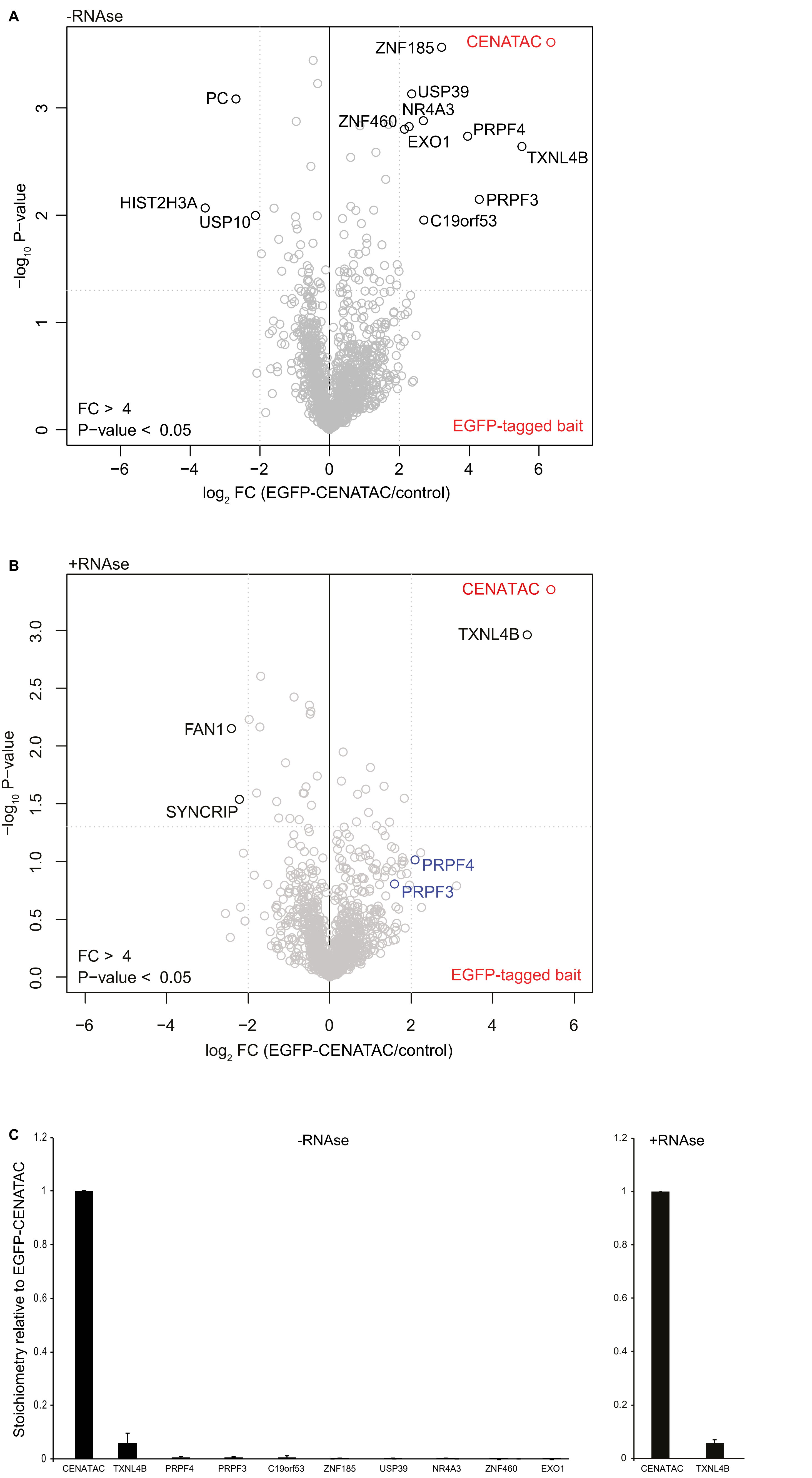

Appendix Figure S7. Volcano plots and stoichiometry of EGFP-CENATAC immunoprecipitations treated with or without RNAse. **A-B)** Mass spec-based identification of interacting proteins with EGFP-CENATAC in EGFP-CENATAC HeLa cell samples treated without **(A)** or with **(B)** RNAse. Statistically enriched proteins in the EGFP-CENTAC pull downs (n = 3), determined using a two tailed t-Test, are depicted on the right-hand side of the volcano plot. The fold change of the LFQ intensities, from the CENTAC over the wt pulldown, is depicted on the x-axis (log2). The y-axis shows the -log10 P-value. Cutoffs are shown with a dotted grey line, with al fold change of 4 and a P-value of 0.05. **C)** Stoichiometry of proteins interacting with EGFP-CENATAC after treating the samples without (left) or with (right) RNAse. The iBAQ value of each proteinis divided by the iBAQ value of CENATAC, and depicted with CENATAC set to 1. Data are shown as mean ± s.d. (n = 3 pulldowns).

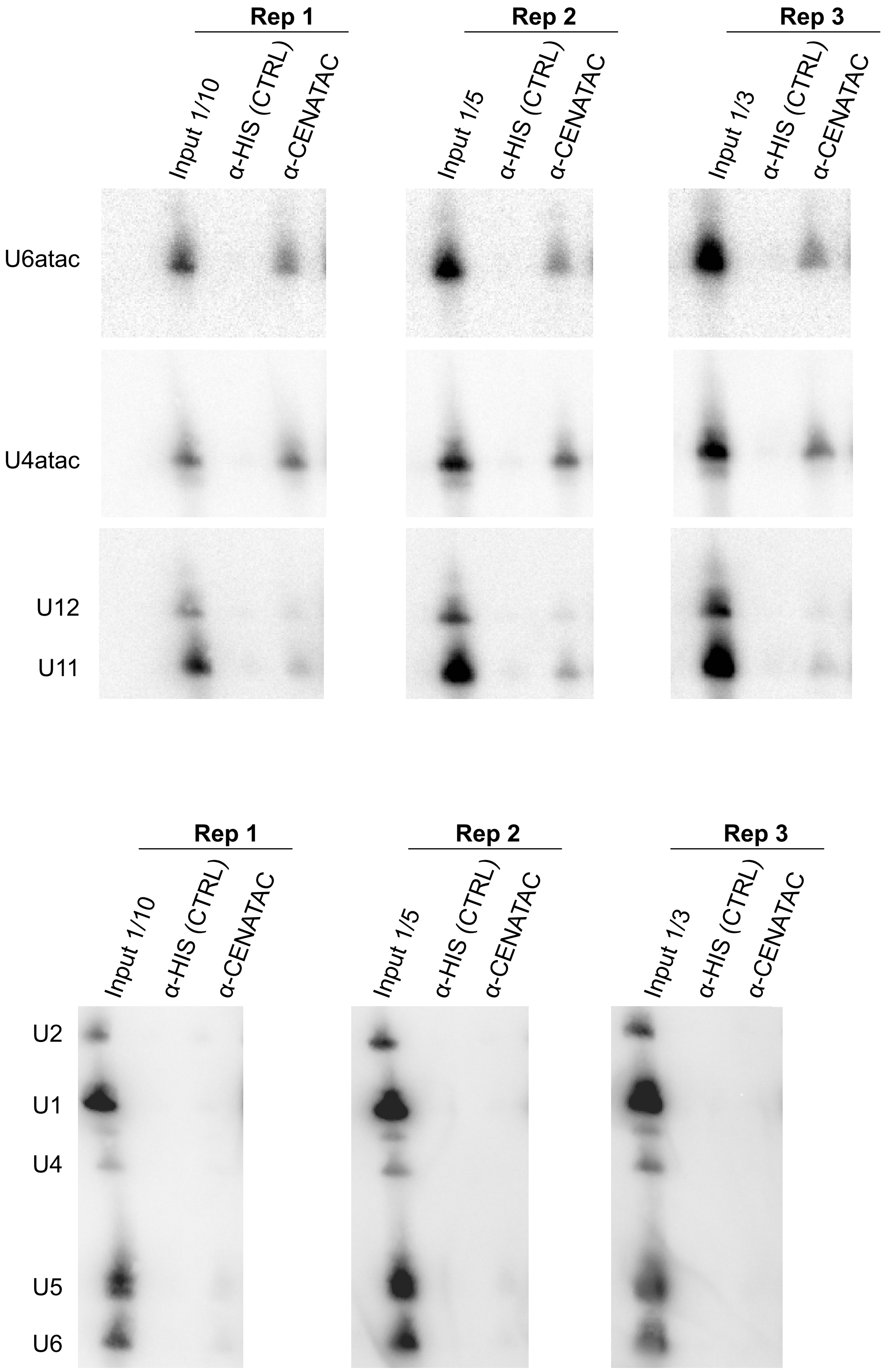

Appendix Figure S8. Northern blots of EGFP-CENATAC immunoprecipitations (N=3) used for the quantification in Fig. 3E.

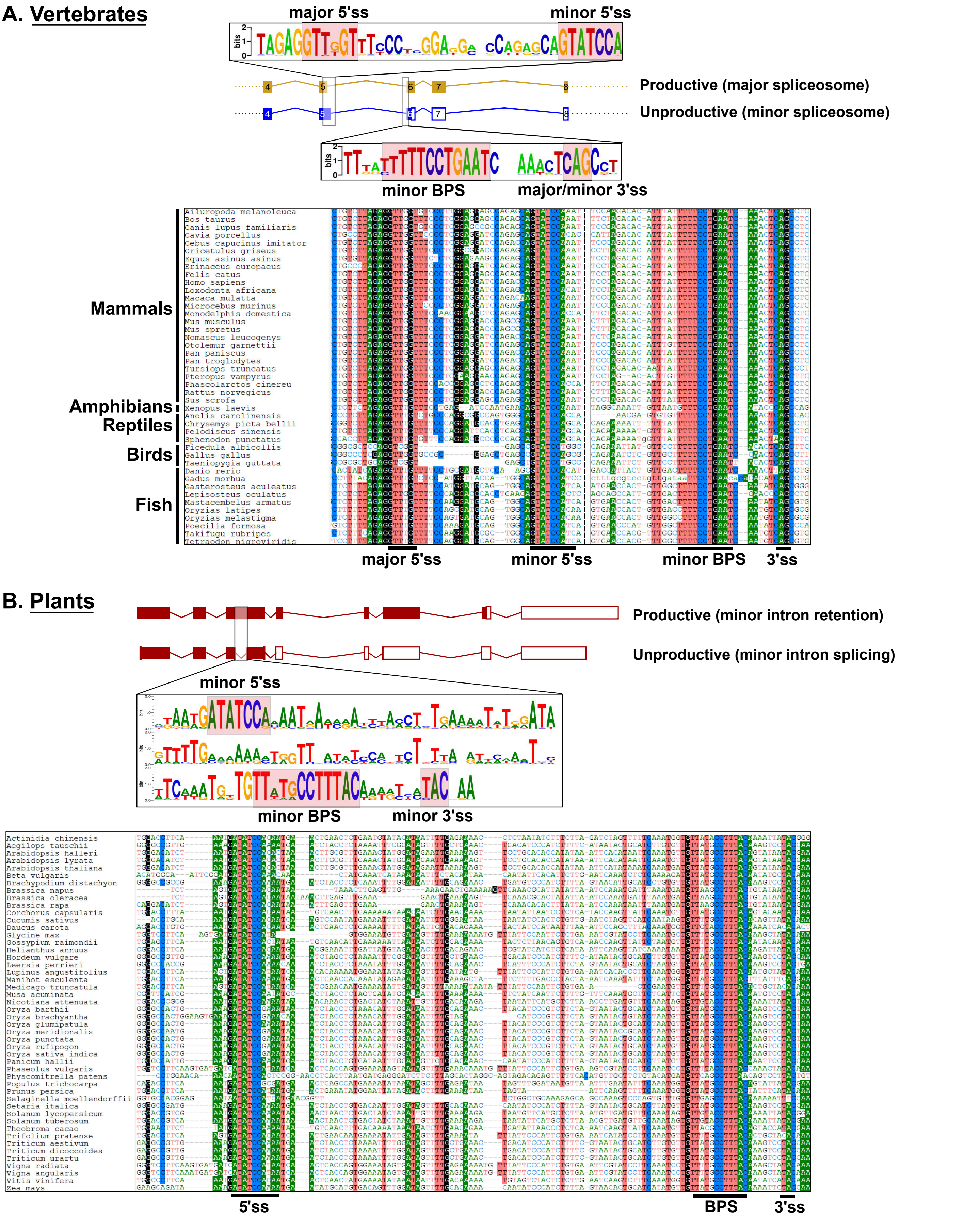

Appendix Figure S9. Evolutionarily conserved sequence elements involved in production of CENATAC productive and nonproductive mRNA isoforms in vertebrates and plants. **A)** In vertebrates splicing of intron 5 by the major spliceosome leads to productive mRNA formation which includes the full-length CENATAC coding sequence. Splicing by the minor spliceosome, however, leads to nonproductive mRNA formation due to introduction of a premature termination codon in exon 6. Sequence logos in the upper panel show the conservation of the major (U2-type) and minor (U12-type) 5'-splice sites, minor BPS and the 3'ss (used by both spliceosomes). The schematic shows exon 4-8 of the human transcripts ENST00000334418.6 (productive isoform) and ENST00000532132.5 (nonproductive isoform). The aligned sequences (lower panel) include 43 vertebrate species (25 mammals, 1 amphibian, 3 reptiles, 1 lizard, 3 birds, 10 fish) that were aligned with DiAlign (dialign.gobics.de) followed by a subsequent sequence logo construction using WebLogo (weblogo.berkeley.edu). **B)** In plants retention of a minor intron embedded in exon 3 leads to productive mRNA formation that includes the full-length CENATAC coding sequence. Splicing of the minor intron by the minor spliceosome leads to a nonproductive mRNA formation due to introduction of premature termination codon in exon 4. The sequence logo shows the conservation of the minor 5'ss, BPS and 3'ss elements within the retained intron. In both panels **A)** and **B)** the filled rectangles indicate protein-coding exons, open rectangles represent non-coding exons. Nucleotides showing at least 85% identity between the species are shaded.

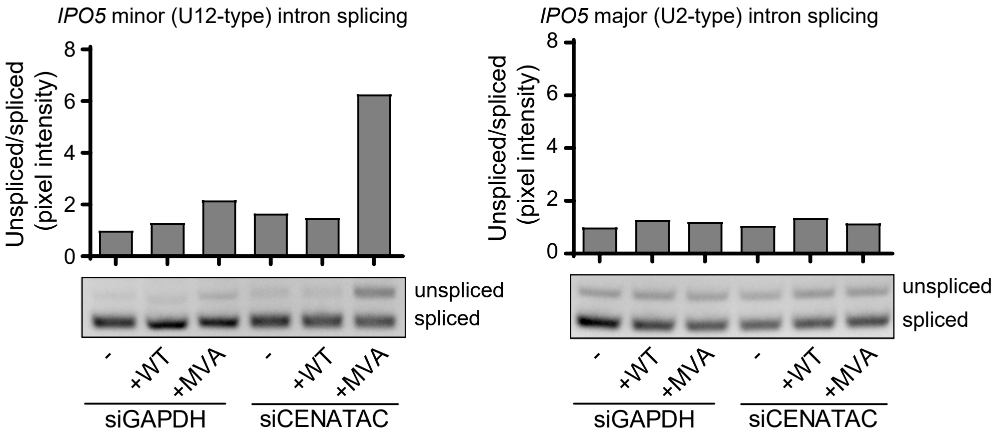

Appendix Figure S10. RT-PCRs and quantification (as in Fig. 4A) of splicing of *IPO5* minor intron 21 and major intron 18 on RNA extracted from DLD-1 cells treated as in Appendix Figure S6. Unspliced/spliced values were normalized to the siGAPDH control.

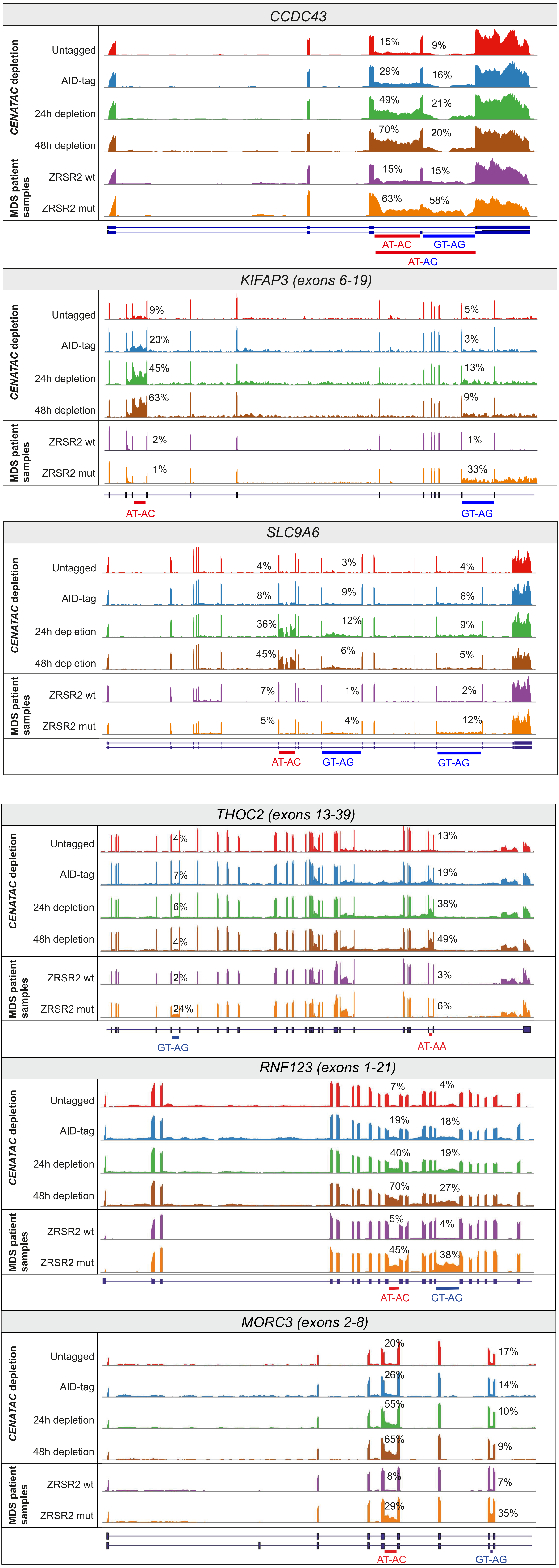

Appendix Figure S11. Sashimi plots showing the effect of CENATAC depletion (24 or 48 hours) in EGFP-AID-CENATAC HeLa cells or *ZRSR2* mutations in the MDS dataset on A- and G-type minor intron retention: AT-AC and GT-AG introns, respectively. Untagged represents the parental unedited HeLa cell line, AID-tag represents the EGFP-AID-CENATAC HeLa cell line; both were depleted of GAPDH for 48h. The AT-AG intron (*CCDC43*, in red and blue) is a hybrid intron that starts from 5' splice site of the AT-AC intron and ends with the 3' splice site of the GT-AG intro.

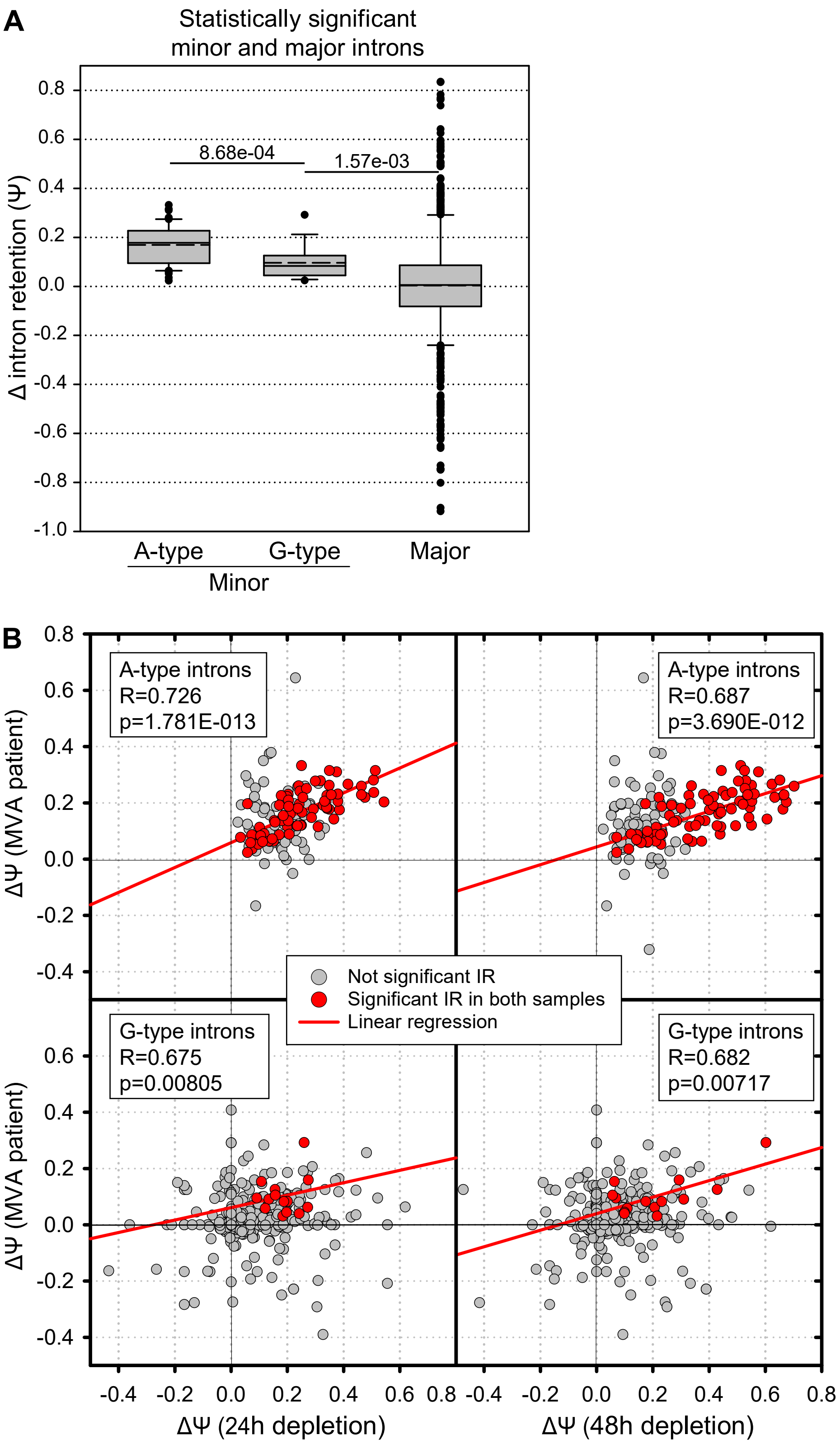

Appendix Figure S12. Analysis of the MVA patient intron retention dataset. A) Comparison of statistically significant minor A-type introns (n= 81), minor G-type introns (n=15), and major introns (n=699). Only introns with on average at least 5 intron-mapping reads were used in the analysis. The boundaries of the boxes indicate 25th and 75th percentiles. Whiskers indicate the 90th and 10th percentiles. Median is indicated with solid line, mean with dashed line inside the box. P values were calculated with Mann-Whitney Rank Sum Tests. **B)** Pairwise comparison of minor intron retention values (ΔΨ) between the MVA datset (y-axis) and the CENTAC depletion datasets (x-axis). Red circles indicate introns with statistically significant intron retention values, while with gray circles the retention values were not statistically significant. For the correlation coefficient calculations only the statistically significant intron retention values (red dots) were used (nonparametic Pearson Product Moment Correlation).

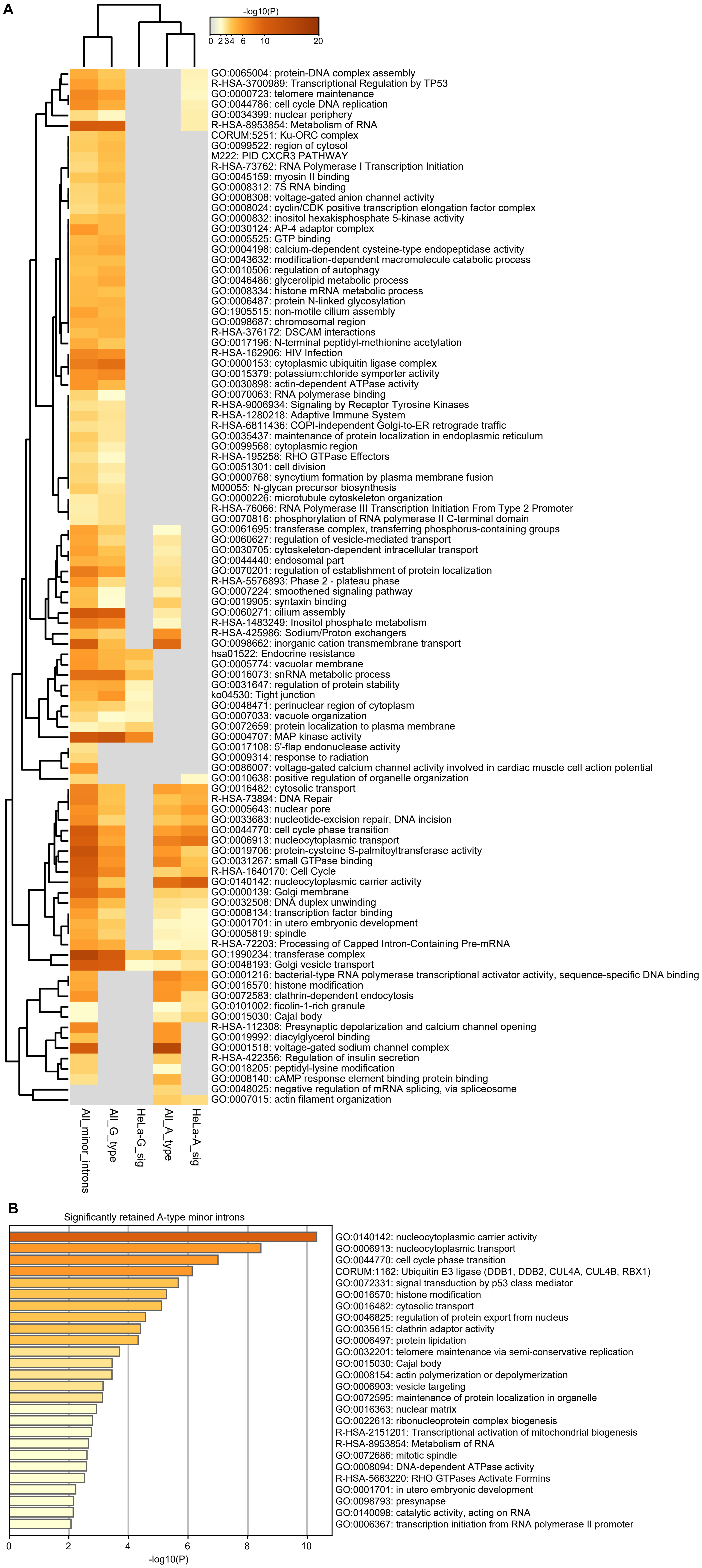

Appendix Figure S13 Metascape analysis (metascape.org) of genes containing significantly retained minor introns following 48h CENTAC depletion. A) Genes with significantly retained A- and G-type introns were analyzed separately (A-type:significant and G-type:significant, respectively) and compared to all genes containing A- or G-type introns (A-type and G-type, respectively) and to all genes containing minor introns. Default parameters with P<0.01 cutoff were used in the analysis. The color scheme indicates the p-values. Top 100 categories are shown. Dataset EV5 includes the numeric p-values related to the figure. Dataset EV6 includes annotations at the gene level and details of the enriched clusters. **B)** GO-term analysis of the genes containing significantly retained A-type introns.
