# Supplementary material for: Chromosomal instability by mutations in the novel minor spliceosome component CENATAC
Source: EMBO J. 2021 May 19;40(14):e106536. doi: 10.15252/embj.2020106536 (PMC8280824; doi:10.15252/embj.2020106536)
Supplement: Supplementary file 2 — Expanded View Figures PDF [file EMBJ-40-e106536-s007.pdf]

Expanded View Figures

ID_5728. The patient was the first child of non-consanguineous healthy Caucasian parents. He had microcephaly, mild developmental delay and mild maculopathy. On last examination at 42 years, his head circumference was 52 cm (-3.5 s.d). His youngest sister had the same clinical features. On last examination at 27 years, her head circumfeı ence was 48 cm (-6 s.d). Neither individual had short stature, dysmorphism or cancer. Both were alive at 47 and 33 years of age, respectively. The siblings also have a healthy sister.

Cytogenetic studies were performed using standard R- and G-banding methods at the 550-band level of resolution. In the proband, five of 68 cells had an abnormal karyotype (47,XYY[1]/47,XY,-1,+4+18[1]/48,XYY,+4[1]/47,XY,+21[1]/45,XY,-22[1]/46,XY[63]. In the proband's sister five of 59 cells had an abnormal karyotype [47,XX,+21[3]/47,XXX-[1]/47,XXX,+18,-22[1]/46,XX[54].

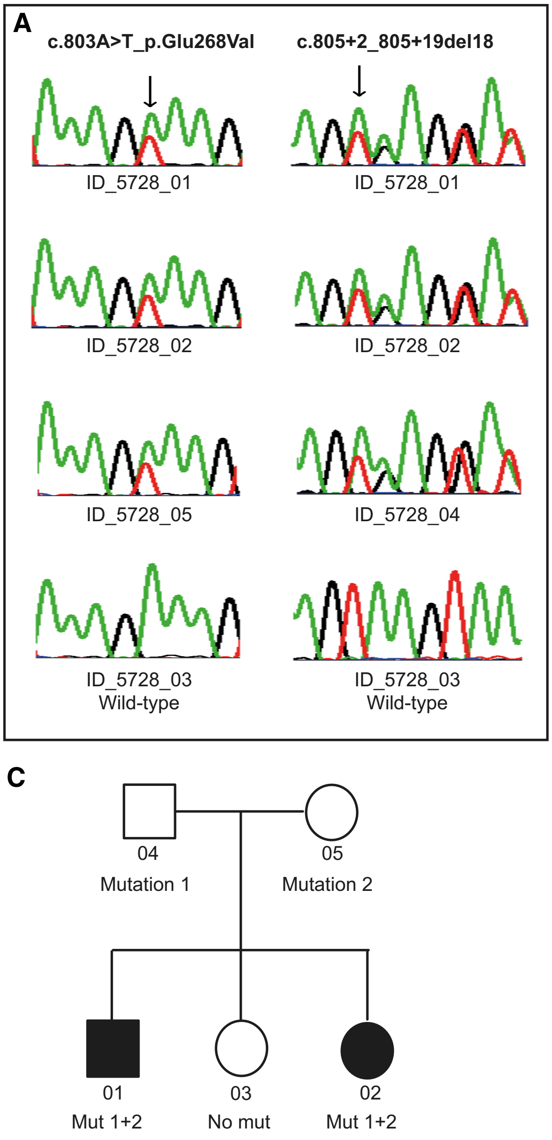

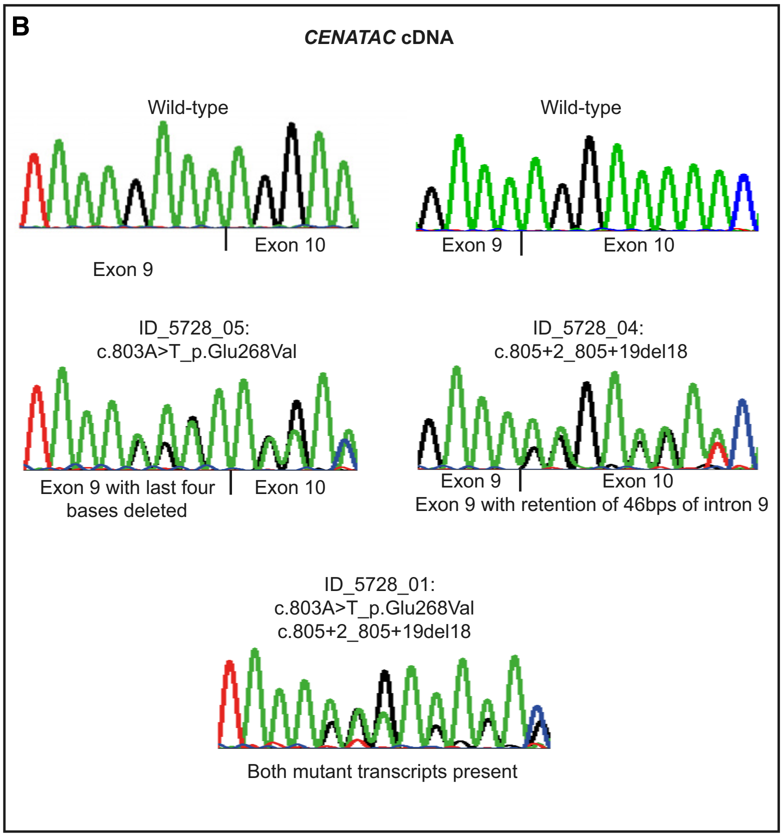

Figure EV1. Case report and chromatograms of individuals with mutations in *CENATAC* (*CCDC84*).

A Sequencing chromatograms showing mutations in blood DNA and corresponding wild-type sequence from a control.

B Sequencing chromatograms from reverse transcription–PCR analysis of RNA showing the effect of *CENATAC* mutations. Maternal cDNA sequencing (ID_5728_05) demonstrates that c.803A>T_p.Glu268Val leads to a translational frameshift as a result of deletion of the last four bases of exon 9. Paternal cDNA sequencing (ID_5728_04) shows that c.805+2_805+19del18 results in retention of 46 bps of intron 9. The affected child's cDNA sequencing (ID_5728_01) demonstrates both mutant transcripts are present.

C Pedigree of family (ID_5728) showing *CENATAC* mutation status.

© 2021 The Authors

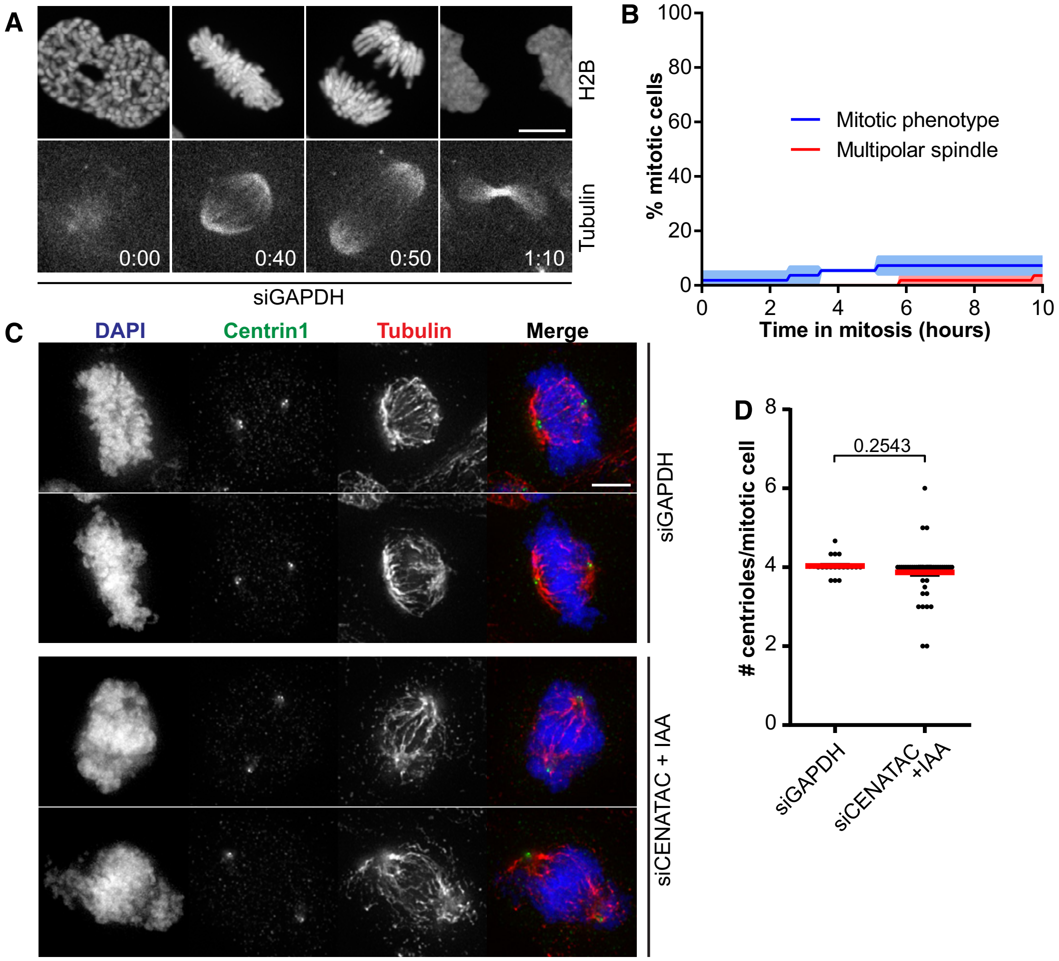

Figure EV2. CENATAC's congression phenotype is not the result of a multipolar mitotic spindle.

A Representative stills of HeLa$^{EGFP\text{-}AID\text{-}CENATAC}$ cells expressing H2B-mNeon and depleted of GAPDH. Microtubules were visualized with SiR-Tubulin. Scale bar, 5 μm. Time in hours. See also Movies EV1 and EV2.

B Quantification of the mitotic phenotype and multipolar spindle formation in time in cells treated as in (A) (three biological replicates, > 44 cells in total).

C Representative immunofluorescence images of HeLa$^{EGFP\text{-}AID\text{-}CENATAC}$ cells depleted of GAPDH or CENATAC and stained with antibodies against Centrin1 and Tubulin. IAA, 3-indoleacetic acid. Scale bar, 5 μm.

D Quantification of the amount of centrioles per mitotic cell treated as in (C) (three biological replicates, > 60 cells in total).

Data information: In (B, D), data are presented as mean ± SEM. *P*-values were calculated with unpaired Student's *t*-tests.

© 2021 The Authors

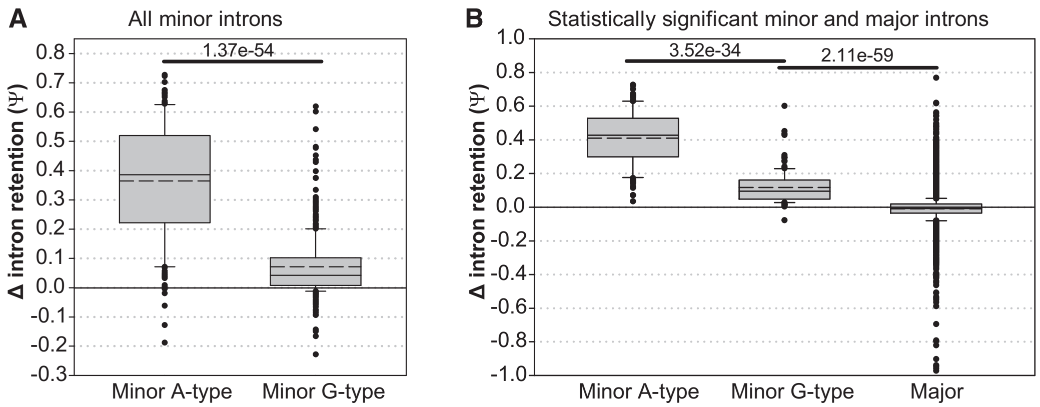

Figure EV3. Comparison of delta-psi values (HeLa$^{EGFP\text{-}AID\text{-}CENATAC}$ cells 48h CENATAC depletion vs. parental cell line 48h GAPDH depletion).

A Comparison of all (statistically significant and not significant) minor A-type (*n* = 179) and minor G-type (*n* = 441) introns (three biological replicates).

B Comparison of statistically significant minor A-type introns (*n* = 133), minor G-type introns (*n* = 130), and major introns (*n* = 8,818; three biological replicates). Only introns with on average at least 5 intron mapping reads were used in the analysis.

Data information: In (A, B), data are presented as median (solid line) and mean (dashed line) inside the boxes. The boundaries of the boxes indicate 25th and 75th percentiles. Whiskers indicate the 90th and 10th percentiles. *P*-values were calculated with Mann–Whitney rank-sum tests.

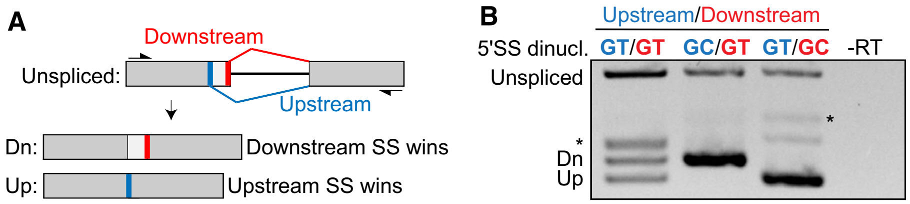

Figure EV4. RT–PCR P120 reporter assay to measure the relative usage of GT-AG and GC-AG G-type minor splice sites in direct competition.

A Schematic diagram showing the overall architecture of the reporter construct with its down- and upstream splice site (thick red and blue bars, respectively) and the products created by splicing (Dn and Up, respectively). SS, splice site.

B RT–PCRs of the reporter with GT-AG or GC-AG splice sites in the down- or upstream positions as indicated above the gel. 5′SS dinucl., 5′SS splice site dinucleotides. *PCR product after use of a cryptic major splice site (not shown in the schematic).

© 2021 The Authors
