# Supplementary material for: Chromosomal instability by mutations in the novel minor spliceosome component CENATAC
Source: EMBO J. 2021 May 19;40(14):e106536. doi: 10.15252/embj.2020106536 (PMC8280824; doi:10.15252/embj.2020106536)
Supplement: Supplementary file 13 — Movie EV2 [file EMBJ-40-e106536-s011.zip › Movie EV2. CENATAC depletion.docx]

**Movie EV2. CENATAC depletion.**

HeLa*^EGFP-AID-CENATAC^* cells expressing H2B-mNeon (upper) and depleted of CENATAC. Microtubules were visualized with SiR-Tubulin (lower). Time in hours.
